# The effect of body mass index and creatinine clearance on serum trough concentration of vancomycin in adult patients

**DOI:** 10.1186/s12879-020-05067-7

**Published:** 2020-05-13

**Authors:** Yuyan Pan, Xiaomei He, Xinyu Yao, Xiaofeng Yang, Fengjiao Wang, Xinyuan Ding, Wenjuan Wang

**Affiliations:** 1 Department of Pharmacy, the Affiliated Changzhou No. 2 People’s Hospital of Nanjing Medical University, Changzhou, 213000 China; 2 Department of Gastroenterology, the Affiliated Changzhou No. 2 People’s Hospital of Nanjing Medical University, Changzhou, 213000 China; 3 Department of neonatology, Children’s Hospital of Soochow University, Suzhou, 215000 China; 4 Department of Pharmacy, Children’s Hospital of Soochow University, Suzhou, 215000 China; 5 Department of Pharmacy, The Affiliated Suzhou Hospital of Nanjing Medical University, Suzhou, 215000 China

**Keywords:** Therapeutic drug monitoring, Vancomycin, Body mass index, Creatinine clearance

## Abstract

**Background:**

The aim of this study was to evaluate the influence of patient body mass index (BMI) and estimated creatinine clearance (CrCl) on serum vancomycin concentrations to define a possible optimal dosage regimen in overweight patients based on data obtained during therapeutic drug monitoring.

**Methods:**

This retrospective study used data collected from January 2017 to January 2019. Adult patients (*n* = 204) received vancomycin treatment at a dose of 1000 mg every 12 h and underwent serum monitoring. Data collected included patient disease category, sex, age, height, weight, vancomycin concentrations, and serum creatinine. The CrCl values were estimated using the Cockcroft-Gault formula. In this study, statistical comparisons were performed on the results of patients according to serum vancomycin concentration.

**Results:**

Serum vancomycin concentration was significantly related to BMI (*P* <  0.001) and CrCl (*P* <  0.05) in adult patients. Furthermore, the trough serum vancomycin concentration showed a logarithmic correlation with BMI (*R* = − 0.5108, 95% CI: − 0.6082 to − 0.3982, *P* <  0.001) and CrCl (*R* = − 0.5739, 95% CI: − 0.6616 to − 0.4707, *P* <  0.001). The multivariate analysis showed that BMI and CrCl are independent contributors to the trough vancomycin concentration. Moreover, some of the patients with higher BMI (≥ 24 kg/m^2^) met the goal trough concentration after an adjustment from 1000 mg every 12 h to 1000 mg every 8 h.

**Conclusions:**

Serum vancomycin concentration decreases progressively with increasing BMI and the augmentation in CrCl in adult patients. The trough concentration of vancomycin should be continuously monitored for patients with a BMI ≥ 24 kg/m^2^, and the dosage regimen should be adjusted to reach the target trough concentration in these patients to reduce the impact of BMI.

## Background

Vancomycin is a glycopeptide antibiotic produced by *Streptococcus orientalis* and has been used for over half a century. Although many antibiotics have been developed and used, vancomycin remains the first-line therapy for invasive multiresistant gram-positive bacterial infections, particularly those involving methicillin-resistant *Staphylococcus aureus* (MRSA) [1]. As the therapeutic range is narrow for vancomycin, therapeutic drug monitoring (TDM) is necessary to maximize clinical efficacy while minimizing the risk of toxicities [2]. We generally plan vancomycin administration using the trough concentration as one measure.

The clinical guidelines recommend always keeping trough vancomycin concentrations at > 10 μg/mL to avoid the development of resistance, and a trough vancomycin concentration of 15–20 μg/mL is recommended for more serious infections [3]. As the elimination of vancomycin relies almost exclusively on glomerular filtration, renal function is one of the most important factors influencing patient exposure to vancomycin [4]. Creatinine clearance (CrCl) is the volume of creatinine in blood plasma cleared per unit time. CrCl is a rapid and effective method for the measurement of renal function. In fact, it is more accurate and provides a better estimation of vancomycin pharmacokinetics to utilize patients’ non-capped CrCl when determining a vancomycin dosing regimen [5].

A large number of studies have shown that CrCl is affected by factors such as age, muscle mass, diet, and proximal tubule secretion of creatinine [6–8]. In fact, early in 2009, Fernando Gerchman et al indicated that a higher body mass index (BMI) rather than body fat distribution was an independent determinant of CrCl in nondiabetic participants [9]. Recently, one study indicated that increased total body weight impacted CrCl and increased the rate of acute kidney injury among patients concurrently treated with piperacillin-tazobactam and vancomycin [10]. Pokorná and colleagues found that vancomycin pharmacokinetics were mainly influenced by actual body weight in neonates [11]. Another study reported that overweight patients have an increased glomerular filtration rate (GFR) and increased renal plasma flow [12]. Thus, the results of these studies indicate that body weight independently affects the filtering capacity of the kidneys and the pharmacokinetic properties of vancomycin. Clinical practice guidelines of the Infectious Disease Society of America (IDSA) recommended a vancomycin dose of 15–20 mg/kg (as actual body weight) every 8–12 h for adult patients with normal renal function, and 15 mg/kg every 6 h in children [13]. Hence, some clinicians increased vancomycin dosing to ≥4000 mg daily. However, larger vancomycin doses are associated with a higher likelihood of vancomycin related nephrotoxicity [14]. Base on the prescription information and the Chinese guidelines, the vancomycin dose and schedule of 1000 mg every 12 h for adult patients are generally used in China. However, such recommendations may be inadequate in overweight patients due to increases in vancomycin clearance and volume of distribution (*V*d) [15]. As a result, a better assessment of renal function and dosage adjustment for vancomycin eliminated by the kidneys is needed in overweight patients [16]. We have treated many patients with relatively higher BMI in whom the serum vancomycin concentrations were found to be moderately lower based on TDM data obtained in our hospital. It is unclear whether a higher BMI increases CrCl, resulting in larger vancomycin dosage requirements. In addition, to date there have been no reports focusing on the effect of both BMI and CrCl on serum vancomycin concentrations. Therefore, the purpose of our study was to determine whether both BMI and CrCl influence trough concentrations of vancomycin in adult patients.

## Methods

### Patients

A single centre retrospective cohort study was conducted at the Affiliated Changzhou No. 2 People’s Hospital of Nanjing Medical University, China. Adult patients ≥18 years of age admitted between January 2017 and January 2019 with suspected or documented Gram-positive infections and receiving empirical vancomycin therapy were recruited. This study was approved by the Ethics Committee of the Affiliated Changzhou No. 2 People’s Hospital of Nanjing Medical University. Patient data were anonymized prior to analysis. Another pharmacist, who was not participating in the study, was responsible for anonymizing these data. The following information including patient demographics such as disease category, sex, age, Acute Physiology and Chronic Health Evaluation (APACHE) II scores, height, weight, and renal function test results such as serum creatinine concentration (Scr), was collected from the patient medical records. The Scr of each patient was obtained before vancomycin administration. Other data collected included vancomycin indication, vancomycin dosing regimens, vancomycin serum trough concentrations, dates, and collection times. Patients who were pregnant, received vancomycin as perioperative antibiotic prophylaxis or on any modality of dialysis were excluded.

### Groups

The prescription information for vancomycin recommends that the drug be used at dosages of 1000 mg every 12 h universally, and used at dosages of 500 mg every 12 h or 1000 mg every 24 h in the elderly population. In fact, all the patients were treated with vancomycin at dosages of 1000 mg every 12 h in this study. As renal function declines with age, elderly patients are more at risk for vancomycin accumulation. Therefore, patients were divided into two groups to rule out the effect of age on the results. Group A, with age < 60 years (*n* = 88 patients), represented the youthful group, and Group B, with age ≥ 60 years (*n* = 116 patients), represented the elderly group. Underweight was defined as a BMI < 18 kg/m^2^, normal weight as a BMI of 18 ~ 23.9 kg/m^2^, overweight as a BMI of 24 ~ 27.9 kg/m^2^, and obesity as a BMI ≥ 28 kg/m^2^. Then, patients were grouped by BMI (< 24 kg/m^2^ and ≥ 24 kg/m^2^) [17] and CrCl (< 90 mL/min and ≥ 90 mL/min) [18].

### Cockcroft-Gault formula: estimated CrCl

CrCl can be estimated using Scr (μmol/L) levels. The Cockcroft–Gault formula is the most widely used clinical method for estimating CrCl (mL/min) to adjust drug dosages [19]. The resulting CrCl is multiplied by 0.85 if the patient is female to correct for the lower CrCl in females. The formulas are as follows:

$$\begin{aligned}&\text{Crc1(Male)} = (140-\text{age}) \times \text{weight(kg)}/ [0.818 \times \text{Scr}(\mu\text{mol/\text{L}})]\\
&\text{Crc1(Famale)}= 0.85 \times (140-\text{age}) \times \text{weight(kg)}/ [0.818 \times \text{Scr}(\mu\text{mol/\text{L}})] \end{aligned}$$

### Measurement of vancomycin

Serum vancomycin concentration was considered to have achieved steady state if ≥3 half-lives (the half-life of vancomycin is 4 ~ 6 h [20]) had elapsed prior to first blood sampling. Dosage adjustment was performed on the subsequent days in patients with higher BMI (≥ 24 kg/m^2^) if the trough vancomycin concentration did not reach the therapeutic range. Vancomycin serum levels were measured using the enzyme multiplied immunoassay technique (EMIT) on a Siemens Viva-E analyser (ELITechGroup B.V., Van Rensselaerweg 46,956 AV, the Netherlands). The linear range for the assay was 2–50 μg/mL, and the limit of quantitation was 2 μg/mL.

### Statistical analysis

Prim 6.0 was used for statistical analyses. Descriptive statistics were processed for all study variables, and continuous variables are expressed as the mean (standard deviation) or median (interquartile range) where applicable. Categorical variables are expressed as numbers (percentages) and were analysed using the chi-squared test or Fisher’s exact test. Linear regression analyses were performed to examine the interaction between steady-state trough concentration and CrCl, trough concentration and BMI. A *P*-value of < 0.05 was considered statistically significant.

## Results

**Fig. 1:**
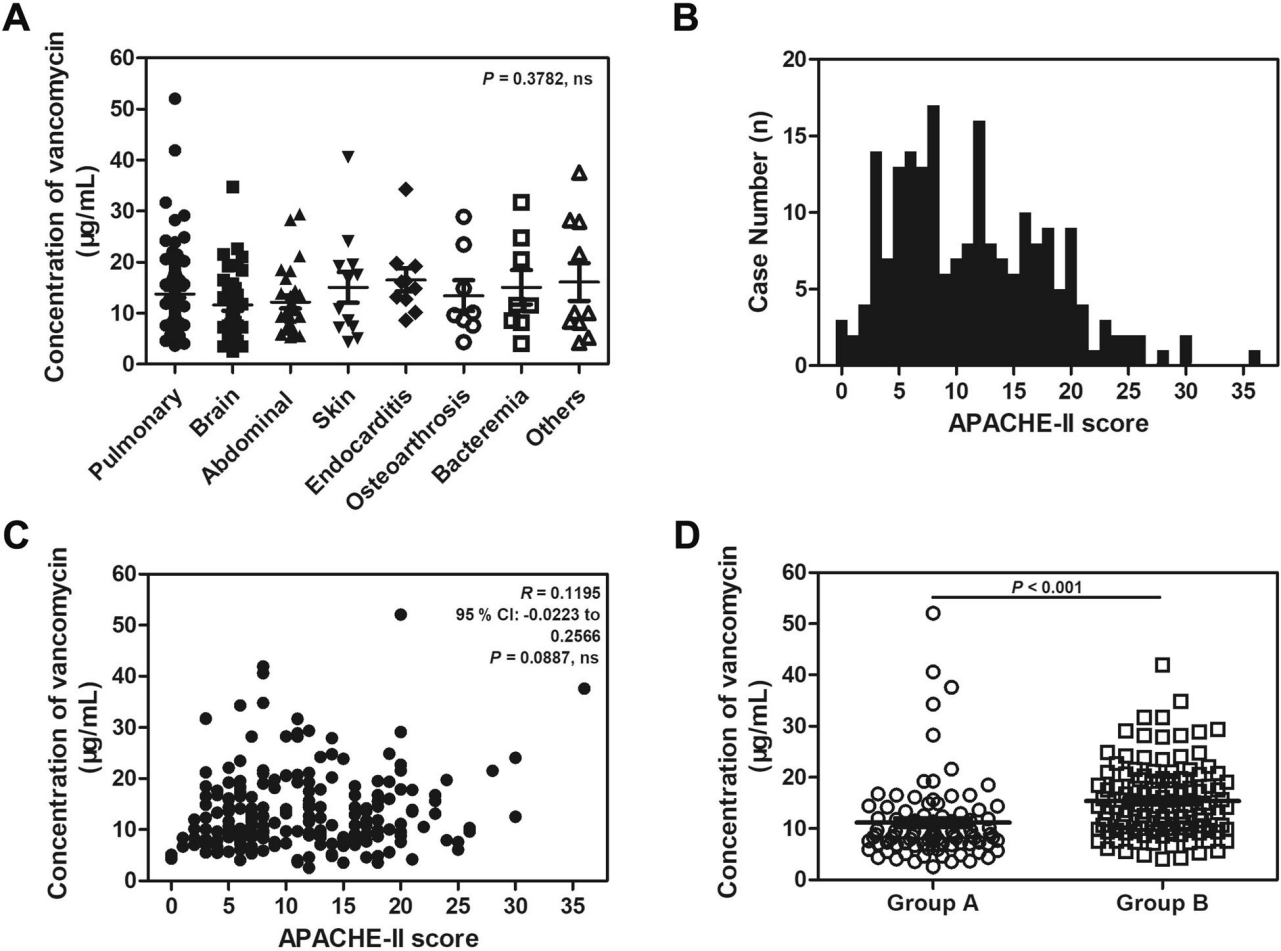
Effects of infectious disease category, APACHE-II score and age on the steady-state trough vancomycin concentration. **a** The correlation between disease categories and trough vancomycin concentration (*P* = 0.3782). **b** The distribution of APACHE-II scores in 204 patients. **c** The correlation between APACHE-II score on admission and trough vancomycin concentration (*P* = 0.0887). **d** The trough vancomycin concentrations in Group A and Group B, the results showed that the trough vancomycin concentrations were lower in Group A than in Group B (*P* < 0.001)

A total of 204 vancomycin serum concentrations were available from all patients, 133 males and 71 female patients. The mean patient age was 60.7 ± 14.8 years, and 65.2% were male patients. The median BMI was 23.8 ± 3.0 kg/m^2^, while the median CrCl was 107.5 ± 57.8 mL/min. There were eight main infectious disease categories among all patients: pulmonary infection (*n* = 94), brain infection (*n* = 36), abdominal infection (*n* = 26), skin and soft tissue infection (*n* = 12), endocarditis (*n* = 10), osteoarthrosis (*n* = 8), bacteraemia (*n* = 8) and other infections (*n* = 10). The pathophysiology of critical illness can cause significant pharmacokinetic changes. As a first step to investigate the effect of infectious disease categories on the trough vancomycin concentration, the statistical analysis showed that there was no significant association of trough vancomycin concentration with disease categories *(P* = 0.3782, Fig. 1a). Moreover, the median (interquartile range) APACHE-II score on admission was 11 (6–16), and there was no significant correlation between the APACHE-II score and the trough vancomycin concentration (*P* = 0.0887, Fig. 1b and c). The result that the patient severity of illness had no effect on the trough vancomycin concentration was confirmed again. As acknowledged, the renal function of elderly patients is likely reduced, and we compared trough vancomycin concentrations between youthful patients and elderly patients. It was found that trough vancomycin concentrations were lower in Group A than in Group B (11.17 ± 8.12 μg/mL *vs* 15.39 ± 7.18 μg/mL, *P* <  0.001, Fig. 1d), which strongly indicated that trough vancomycin concentration might be correlated with age. It is clearly that age is a risk factor for reduced renal function which in turn vancomycin concentration is affected by age. To better understand whether the trough vancomycin concentration was mediated by CrCl or BMI, we evaluated these two groups to eliminate the effect of age. According to the clinical guidelines of trough vancomycin concentration, a trough vancomycin concentration of < 10 μg/mL was assigned to low concentration and a trough vancomycin concentration of > 20 μg/mL was considered a high concentration. As shown in Table 1, the vancomycin concentration in Group A (age < 60 years) was significantly related to BMI (*P* <  0.001) and CrCl (*P* = 0.0162). However, there was no significant association of vancomycin concentration with sex. These parameters mentioned above showed the same tendencies in the elderly population composing Group B (age ≥ 60 years). These results suggest that the trough vancomycin concentration was significantly affected by BMI and CrCl in both youthful patients and elderly patients.

**Table 1:** The correlation between serum vancomycin concentrations and characteristics of patients included in different groups

Groups	Demographics and characteristics	Case no.	Vancomycin concentration (μg/mL)			Chi-square	*P*-value
			< 10	10 ~ 20	> 20		
Group A (age < 60 years)	Sex					1.443	0.4861
	Male	61	40	18	3		
	Female	27	15	9	3		
	BMI (kg/m^2^)					22.450	< 0.001^***^
	< 24	36	12	19	5		
	≥ 24	52	43	8	1		
	CrCl (mL/min)					8.247	0.0162^*^
	< 90	21	9	8	4		
	≥ 90	67	46	19	2		
Group B (age ≥ 60 years)	Sex					3.206	0.2013
	Male	72	23	35	14		
	Female	44	8	23	13		
	BMI (kg/m^2^)					25.360	< 0.001^***^
	< 24	82	11	48	23		
	≥ 24	34	20	10	4		
	CrCl (mL/min)					18.320	0.0001^***^
	< 90	74	10	43	21		
	≥ 90	42	21	15	6		

*P* by Chi-square test, * *P* < 0.05, *** *P* < 0.001 were considered significant

**Fig. 2:**
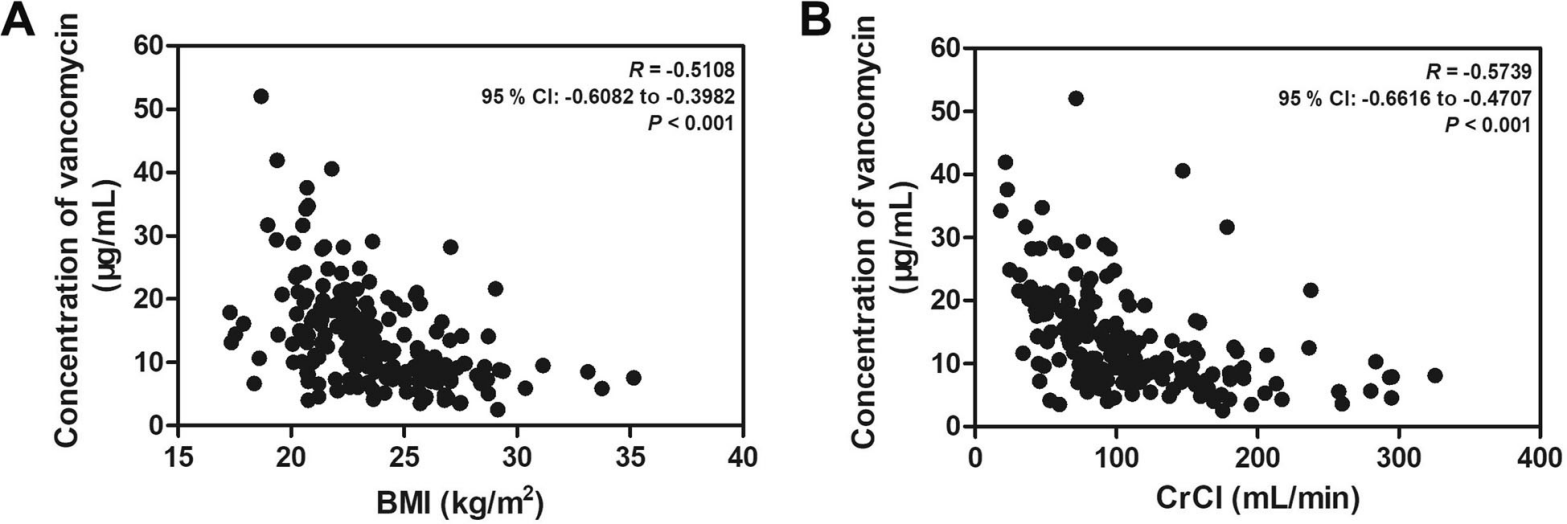
The relationships between steady-state trough vancomycin concentration and BMI and CrCl were linear. The serum vancomycin concentration showed a logarithmic correlation with BMI (*R* = −0.5108, 95% CI: −0.6082 to −0.3982, *P* < 0.001, **a**) and CrCl (*R* = −0.5739, 95% CI: −0.6616 to − 0.4707, *P* < 0.001, **b**) in all adult patients included in our study

Simultaneously, we further analysed the correlation between CrCl and trough vancomycin concentration, as well as the correlation between BMI and trough vancomycin concentration. All adult patients who received vancomycin at dosages of 1000 mg every 12 h are shown in Fig. 2. As BMI and CrCl increased, the percentage of patients with adequate serum concentrations decreased. Most notably, trough serum vancomycin concentration showed a logarithmic correlation with BMI (*R* = − 0.5108, 95% confidence interval (CI): − 0.6082 to − 0.3982, *P* <  0.001, Fig. 2a) and CrCl (*R* = − 0.5739, 95% CI: − 0.6616 to − 0.4707, *P* <  0.001, Fig. 2b) in all adult patients included in this study. We further classified BMI as normal (BMI <  24 kg/m^2^) and overweight (BMI ≥ 24 kg/m^2^) [17], CrCl as high (CrCl ≥90 ml/min) and low (CrCl < 90 ml/min) [18]. We also classified trough vancomycin concentration as high and low based on the median cut-off value. The multivariate analysis showed that both BMI and CrCl were significantly related to trough vancomycin concentration (Table 2). The overweight patients showed lower vancomycin concentration (*OR* = 0.171, 95% CI 0.087 to 0.337, *P* < 0.001), and the patients with low CrCl had higher vancomycin concentration (*OR* = 3.425, 95% CI 1.770 to 6.623, *P* < 0.001). These results indicated that both BMI and CrCl are independent contributors to the trough vancomycin concentration.

**Table 2:** The multivariate analysis of BMI and CrCl related to trough vancomycin concentration

Comparison	Variable	OR	95% CI	*P*-value
Vancomycin concentration (High *vs* low)	BMI, kg/m^2^			
	Normal (< 24)	Reference	0.087 ~ 0.337	< 0.001^***^
	Overweight (≥ 24)	0.171		
	CrCl, ml/min			
	High (≥ 90)	Reference	1.770 ~ 6.623	< 0.001^***^
	Low (< 90)	3.425		

*** *P* < 0.001 was considered significant

In addition, the dosage schedule of patients (*n* = 24) with higher BMI (≥ 24 kg/m^2^, 26.88 ± 1.71 kg/m^2^) in this study was adjusted and the time required to achieve a new steady-state was calculated. After an adjustment from 1000 mg every 12 h to 1000 mg every 8 h, the trough concentration in three patients increased from 4.00 to 20.30 μg/mL, 7.32 to 21.34 μg/mL and 5.08 to 24.31 μg/mL, while the serum concentrations achieved the goal trough concentration in 87.5% (*n* = 21) of these patients.

## Discussion

Vancomycin pharmacokinetics is highly variable even among patients with similar characteristics. In recent years, the way in which to improve the clinical efficacy of vancomycin and reduce its side effects has become the focus of clinical research. However, serum vancomycin concentrations often do not reach the target treatment level in overweight patients [21]. The World Health Organization (WHO) has published a standard for evaluating overweight and obesity in adults based on BMI [22], and there are also standard BMI values in China [17].

At present, the Chinese standard BMI cut-off points are ≥24 kg/m^2^ for overweight and ≥ 28 kg/m^2^ for obesity. It has been reported that the thresholds of BMI may be different by age group [23]. In this study, patients were divided into two groups to rule out the effect of age. Our study found that the steady-state trough vancomycin concentration was negatively correlated with BMI and CrCl. Moreover, the elderly patients also showed a significant tendency with the same dosing regimen. In addition, we observed a significant logarithmic correlation between the steady-state trough concentration and BMI, and the steady-state trough concentration and CrCl. The serum vancomycin concentration was strongly associated with BMI and CrCl in all adult patients. According to our study, BMI and CrCl were both independent factors for trough vancomycin concentration. Vancomycin is a glycopeptide with hydrophilic properties, which is one of the key antibiotics used empirically in patients to treat organisms such as MRSA, enterococcus and other resistant gram-positive bacteria. The obese patients who take hydrophilic drugs have an increased *V*d and therefore a decreased serum concentration [24]. Low vancomycin concentration can lead to treatment failure, so it is necessary to study the correlation between BMI and the concentration of vancomycin. Indeed, there were studies already had shown that *V*d was associated with BMI, and drug clearance (CL) had a positive correlation with CrCl [25, 26]. Hence, we speculate that the effect of BMI on vancomycin concentration may be achieved by affecting the *V*d, while the effect of CrCl on vancomycin concentration was mainly achieved by affecting the drug CL. Based on it, the vancomycin dosage regimen should be adjusted in a timely manner to reach the target trough concentration by considering of CrCl as well as BMI. In fact, we further adjusted the vancomycin dosage schedule (1000 mg every 8 h) in 24 patients with BMI ≥ 24 kg/m^2^, most of whom successfully achieved the goal trough concentration.

However, this study has several limitations to consider. The main limitation of the study was that it was not a strictly controlled clinical trial but a retrospective investigation with data produced in clinical settings. Other limitations must also be considered, including the small sample size. We also did not take into account the physical condition of the patients, such as a serious illness throughout their entire hospitalization. Last, we did not evaluate the clinical outcomes or adverse drug reactions of the patients.

In this study, we tried to adjust the dosage regimen for patients with BMI ≥ 24 kg/m^2^, and the effects were mostly ideal. Therefore, the combination of BMI, CrCl and serum vancomycin concentration monitoring should play an important role in dosage adjustment in individual patients.

## Conclusions

Our results suggest that serum vancomycin concentration decreases progressively with increasing BMI and the increase in CrCl in adult patients. Therefore, dose adjustment should be based on BMI and CrCl for safe and effective use of vancomycin in adult patients. The trough concentration of vancomycin should be continuously monitored for patients with BMI ≥ 24 kg/m^2^. Further research to confirm this negative association is needed.

## Data Availability

The datasets used and/or analyzed during the current study are available from the corresponding author on reasonable request.

## Abbreviations

APACHE: Acute Physiology and Chronic Health Evaluation
BMI: Body mass index
CI: Confidence interval
CL: Clearance
CrCl: Creatinine clearance
EMIT: Enzyme multiplied immunoassay technique
GFR: Glomerular filtration rate
IDSA: Infectious Disease Society of America
MRSA: Methicillin-resistant *Staphylococcus aureu*
Scr: Serum creatinine concentration
TDM: Therapeutic drug monitoring
*V*d: Volume of distribution
WHO: World Health Organization
